# Preparation of Cross-Linked Graphene Oxide on Polyethersulfone Membrane for Pharmaceuticals and Personal Care Products Removal

**DOI:** 10.3390/polym12091921

**Published:** 2020-08-26

**Authors:** Yanyan Lou, Fibor J. Tan, Rong Zeng, Mengen Wang, Pan Li, Shengji Xia

**Affiliations:** 1 State Key Laboratory of Pollution Control and Resources Reuse, Tongji University, Shanghai 200092, China; louyanyan@tongji.edu.cn (Y.L.); Margaret.Zeng@outlook.com (R.Z.); dejyszlt@126.com (M.W.); lipan@tongji.edu.cn (P.L.); 2 School of Civil, Environmental, and Geological Engineering, Mapua University, Manila 1002, Philippines; FJTan@mapua.edu.ph; 3 Yuchengco Innovation Center, Mapua University, Manila 1002, Philippines; 4 Shanghai Institute of Pollution Control and Ecological Security, Shanghai 200092, China; 5 Key Laboratory of Yangtze River Water Environment, Ministry of Education, Tongji University, Shanghai 200092, China

**Keywords:** graphene oxide, polyethersulfone, cross-linked, ultrafiltration membrane, PPCPs

## Abstract

The unique two-dimensional structure and chemical properties of graphene oxide (GO) provide a convenient method for preparing novel membranes. In this study, GO membranes were prepared through filtration by a pressure-assisted self-assembly method involving the cross-linking of three diamine monomers on a polyethersulfone (PES) support. The different small molecular diamines, ethylenediamine, butanediamine, and p-phenylenediamine, were introduced as cross-linking agents to investigate the effect of diamine on the properties of GO membranes. The hydrophobic substances ibuprofen, gemfibrozil, and triclosan were selected as target pharmaceuticals and personal care products (PPCPs). The adsorption and molecular sieving activities of PPCPs by cross-linked GO membranes at a pH of 3 were investigated. The permeate water was analyzed for dissolved organic carbon, ultraviolet absorption at 254 nm, molecular weight distribution, and fluorescence excitation–emission matrices. The results showed that the removal of hydrophobic PPCPs by GO membranes was mainly due to their adsorption and molecular sieving activities. Adsorption was mainly determined by the hydrophilic and hydrophobic properties of the membranes and PPCPs. The interception effect was mainly determined by the interlayer spacing between the GO membranes and the molecular weight and steric hindrance of the PPCPs. A smaller spacing of the GO membrane layers resulted in greater steric hindrance and a higher removal rate.

## 1. Introduction

With the rapid development of the economy, the problems of water shortage and pollution have become increasingly prominent. Among them, pharmaceuticals and personal care products (PPCPs), which are widely found in natural water bodies, have attracted increasing attention [1,2]. PPCPs are an emerging pollutant that are common in daily life. However, PPCPs are not completely metabolized in and/or are improperly disposed of in the environment, such as in sewage treatment and in bodies of water. This leads to serious pollution by PPCPs in water environments [1,2]. Typical PPCPs include antibiotics, estrogens, anti-inflammatory analgesics, synthetic musk, and bactericidal cleansers and disinfectants. Some studies found that nearly 94% of PPCPs were toxic to microorganisms, plants, animals, and humans [3].

Among these PPCPs, ibuprofen, gemfibrozil and triclosan are the most commonly detected drugs in the water environment. Ibuprofen is a non-steroidal anti-inflammatory drug; it has been found to exist in sewer wastewater in significant quantities up to 10 and 169 μg/L. The presence of Ibu in nature can have traumatic effects on living organisms. The combination of Ibu with other drugs can stop cell reproduction in human embryos, and some reports show that contact with Ibu can have a negative effect on aquatic vertebrate reproduction [4]. Gemfibrozil has been found in surface waters across Europe and the United States [5], at concentrations as high as 1.55 mg/L [6]. As a lipid regulator, gemfibrozil is used to reduce plasma triglycerides and total cholesterol, and to increase high-density lipoproteins [7]. Several effects of gemfibrozil have been reported on aquatic organisms, particularly fish. For instance, alteration in lipoproteins composition was observed in rainbow trout (Oncorhynchus mykiss), which may result in a reduction in the nutritional quality of the organisms [8]. Triclosan, an endocrine disruptor, was banned by the USA’s Food and Drug Administration (FDA 2016) in September 2016. However, its numerous residue in environments still has potential threats to ecological safety and human health [9]. Some studies revealed that triclosan has membranotropic effects, acts as a mitochondrial uncoupler in mammalian cells, and even triggered a decrease in fish sperm counts [10,11].

Removal of the above-mentioned emerging pollutants is deemed to be a significant concern in protecting the environment and preventing potential adverse effects to living organisms. Traditional water treatment processes, however, are unable to effectively remove PPCPs. As an alternative technology, the use of membranes has attracted attention due to its wide range of removal, low energy consumption, compact equipment, and simple operation [12,13]. One of the membrane separation processes, nanofiltration (NF) is a good means of removing PPCPs [14,15]. However, in practical applications, the traditional NF membrane does not simultaneously provide good chemical stability, microbial resistance, and high mechanical strength. Recently, some studies on the use of nanomaterials for membrane modification have generated great interest, such as carbon nanotube [16,17,18,19], graphene oxide [20,21,22,23] and molybdenum disulfide [24,25,26,27].

Adsorption, as in nanofiltration, is one of the most promising and effective treatments for the removal of different types of micropollutants if the adsorbent is reusable [28,29]. In recent years, graphene has become a popular material, attracting attention in the fields of chemistry, physics, and materials science because of its optimal electronic, thermal, and mechanical properties [30,31,32,33]. Graphene oxide (GO) is a derivative of graphene, but they have differing properties. A new generation nanomaterial, sheets of GO, possess numerous oxygen-containing functional groups: hydroxyl and carboxyl groups are located around the edges, whereas carbonyl and epoxide groups are in the center [34,35]. These active groups can be used to induce chemical reactions and provide GO with additional functional groups after the modification, thereby increasing the flexibility and diversity of GO applications. In addition, GO has the advantages of chargeability, a large specific surface area, high strength, and antibacterial properties. Moreover, GO easily disperses in an aqueous solution and organic solvents. All these properties may be valuable for application in composite membrane materials [36]. The most common method to modify the separation membrane is by adding GO to the casting membrane materials for phase inversion [37]. Some studies show that the hydrophilicity of the modified membrane is enhanced, and the water flux is increased while ensuring the removal rate of contaminants [38,39,40,41].

In the fabrication of desalination membranes, GO could also be applied to freestanding membranes, on the surface of membranes, which utilizes GO as a separation layer directly [42]. The GO layers may be controlled by introducing a high-molecular polymer, metal nanoparticles, electrolytes, inorganic particles, and other substances [26,43].

To date, GO-based membranes have been studied mainly for desalination and dye removal; however, there are only a few papers focusing on the PPCP removal by GO films. In this study, a cross-linked GO film was prepared by the self-assembly method. The polyethersulfone (PES) support was pretreated with dopamine, followed by the introduction of different small molecular diamines, including ethylenediamine (EDA), butanediamine (BDA), and p-phenylenediamine (PPD), as cross-linking agents to investigate the effect of diamine on the properties of GO membranes. The hydrophobic substances ibuprofen, gemfibrozil, and triclosan were selected as target PPCP compounds. The adsorption and molecular sieving of PPCPs by cross-linked GO membranes were investigated.

## 2. Materials and Methods

### 2.1. Materials

Humic acid was purchased from Sigma–Aldrich (St. Louis, MO, USA). PES ultrafiltration membrane (PBVK07610; Millipore Corporation, Bedford, MA, USA) were used as supports for the GO film. Graphene oxide sol (1 wt %, purity > 99%), dopamine hydrochloride (purity > 98%), tris(hydroxymethyl)aminomethane acetic acid (purity > 99%), methanol (CH_3_OH, purity > 99%), EDA, BDA, PPD, and tannic acid were obtained from Aladdin Industrial Co. (Shanghai, China). HCl and NaOH were purchased from Sinopharm Group Chemical Reagent Co. Ltd. (Shanghai, China). Ibuprofen, gemfibrozil and triclosan, the target PPCP compounds were all purchased from Aladdin Industrial Co., China. The details are shown in Table 1.

### 2.2. Fabrication of the Membranes

#### 2.2.1. Pretreatment of Support Layer

PES ultrafiltration membrane (Millipore Corporation, USA) was used as a support. The membrane surface was washed before use. A mixed solution containing 2 g/L dopamine hydrochloride and 10 mM of Tris(hydroxymethyl)aminomethane acetic acid was prepared, and the support PES ultrafiltration membrane was immersed in the mixed solution for 48 h.

#### 2.2.2. Preparation of Cross-Linked GO Self-Supporting Film

A dispersion solution is prepared by diluting 1 wt % GO sol and small molecule binary amines (EDA, BDA, PPD) were added to form a 400 ppm GO solution containing 0.1 M amine monomer, which was sonicated for 20 min to form a uniform dispersion. The dispersion solution was set aside for 5 h. After the amine monomer was sufficiently reacted with GO, a certain amount of the dispersion solution was diluted to a volume of 100 mL. This solution was filtered onto a PES ultrafiltration membrane (GO surface density 25 mg/m^2^) under a pressure of 1 bar by a filtration of self-assembly method. The cross-linked GO film was dried at 65 °C for 2 h to promote the cross-linking reaction. The prepared cross-linked GO film was immersed in methanol for 12 h to remove unreacted excess diamine monomer. The film was removed and left to dry at room temperature for later use.

### 2.3. Membrane Characterization

#### 2.3.1. Morphology and Microstructure

In the cross-linked GO membrane, the interlayer spacing is an important part of the membrane structure, and its size corresponds to the “pore diameter” of the GO membrane. In this experiment, the interlayer spacing was measured by X-ray diffractometer (XRD) using a model D8 Advance (Bruker Corporation, Karlsruhe, Germany) equipped with a Cu target Kα radioactive source. In order to investigate the condition of the membrane under pressure, XRD was used to determine the change in the interlayer spacing of the membrane under pressure filtration. XRD was used at 40 kV and 40 mA to reveal the microstructure and crystallization properties of the GO membranes. The layer spacing of the GO membranes can be calculated by the Bragg Equation (1)

(1) λ=2dsinθ

where *λ* is the wavelength of the Cu X-ray beam (0.15406 nm), *d* is the interlayer spacing of GO membranes, and *θ* is the diffraction angle.

The top surface and cross-section micromorphology of the prepared membranes were observed by field-emission scanning electron microscopy (FE-SEM), using a Phenom Pro device (Phenom-World Company, Eindhoven, The Netherlands). FE-SEM was performed under standard high-vacuum conditions at 5.00 kV and the samples were photographed to obtain micromorphology images of each membrane. Before analysis, the samples were air-dried and sputter-coated with a thin gold layer to enhance electrical conductivity.

#### 2.3.2. Chemical Properties

The surface chemistry and chemical compositions of the GO membranes were analyzed by attenuated total reflectance-Fourier transform infrared spectroscopy (ATR–FTIR) using a Nicolet iS5 apparatus (Thermo Fisher Scientific Inc., Waltham, MA, USA) and X-ray photoelectron spectroscopy (XPS) using a PHI-5300 device (PerkinElmer Inc., Waltham, MA, USA). The ATR–FTIR analysis were performed using a Ge crystal as a background with a wavenumber range of 800–4000 cm^−1^ to analyze the change in membrane structure and functional groups. XPS with a monochromatic Al Kα X-ray source was conducted at 14 kV and 250 W to determine the composition of the elements.

#### 2.3.3. Surface Hydrophilicity

The dynamic water contact angle was measured using an optical tension meter (Attension Theta Lite; Biolin Scientific Co. Ltd., Gothenburg, Sweden) to assess the surface hydrophilicity of membranes. The trigger function in OneAttension software was used to automatically record experimental data. After the data recording was completed, the Analysis interface was used for data analysis. The samples were dried at room temperature for 24 h before the test. A drop of pure water (approximately 0.05 mL) was dispensed onto the dry flattened membrane surface using a micro-stainless-steel syringe. The instrument software was set to record data at 60 FPS for 5 s. A reliable contact angle value was obtained by averaging three measurements from different points on membrane surfaces to minimize error.

#### 2.3.4. Filtration Experiments

**Figure 1:**
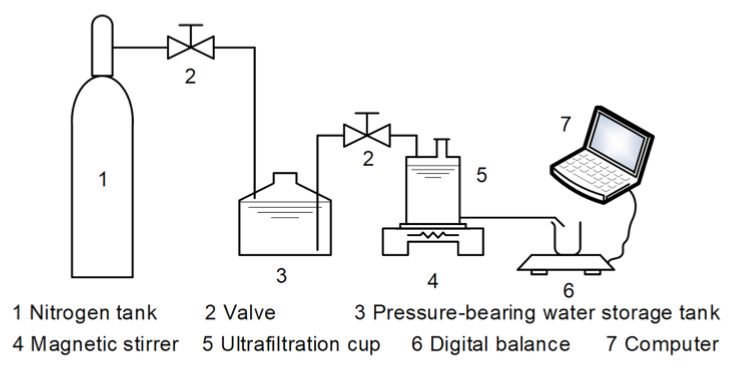
Schematic diagram of the experimental setup.

The permeation and PPCPs removal performance of the prepared GO membranes were investigated using a dead-end filtration system. The system consisted of a compressed nitrogen bottle, digital balance connected to a computer, 5-L dispensing pressure vessel, and an ultrafiltration cup with an effective filtration area of 13.85 cm^2^. The whole device is shown in Figure 1. Before the experiment, the membranes were washed with pure water at 3 bar for 5 h to achieve stable water flux. The water flux was calculated by using the following Equation (2)

(2) $$J_{W}=\frac{Q}{AP\Delta t}$$

where *J_W_* is the water flux (L/m^2^·h·bar), *Q* is the volume of the permeate water (L), *A* is the effective membrane area (m^2^), *P* is filtration pressure (bar), and Δ*t* is the permeation time (h).

#### 2.3.5. Instruments and Methods Used for Water Analysis

Ultra-high-performance liquid chromatography was performed using an ACQUITY UPLC device (Waters Corporation, Milford, MA, USA) to analyze the concentration of the target PPCP compounds (ibuprofen, gemfibrozil, and triclosan), followed by ultraviolet detection. The mobile phase consisted of a mixture of acetonitrile and 0.1% formic acid solution. A total organic carbon analyzer (TOC-L CPH; SHIMADZU Corporation, Kyoto, Japan) was used to measure the dissolved organic carbon contents in each sample. A Lengguang-UV765 ultraviolet-visible (UV–Vis) scanning spectrophotometer (Shanghai INESA Co. Ltd., Shanghai, China) with a glass cuvette (diameter of 1 cm) was used to measure the absorbance of each sample at 254 nm. The UV_254_ value reflects the amount of humic macromolecular organics in water and aromatic compounds containing C=C double bonds and C=O bonds. Fluorescence excitation–emission matrices (EEMs) were collected using a Cary Eclipse fluorescence spectrophotometer (Agilent Technologies Co. Ltd., Santa Clara, CA, USA). EEMs can be used to detect the composition and content of organic matter in water efficiently and conveniently. The scanning range of excitation and emission wavelength was from 200 to 600 nm, and the step length was 10 nm.

## 3. Results and Discussion

### 3.1. Characterization of Cross-Linked GO Self-Supporting Membranes

**Figure 2:**
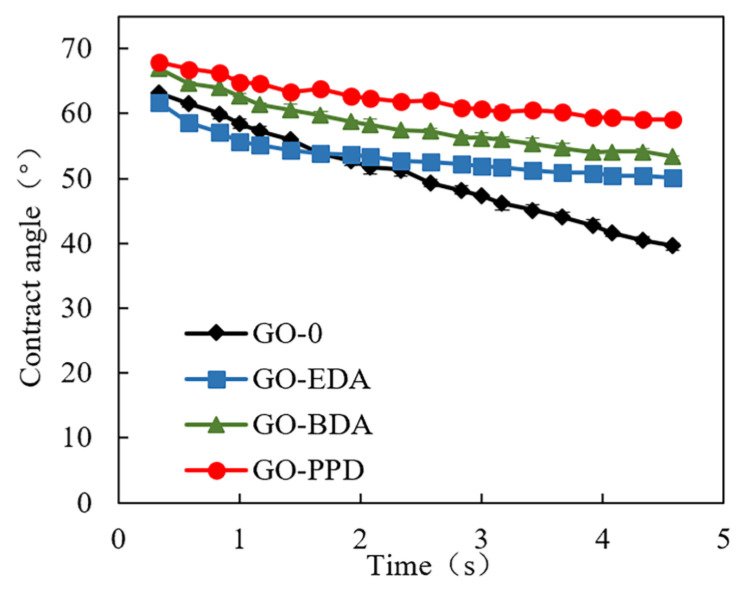
Dynamic contact angle of the various cross-linked GO membranes for 5 s.

Although the initial contact angle of GO-0 was large (63.1°), it decreased rapidly (39.7°) (Figure 2). This is because GO-0 has a large number of hydrophilic groups, including hydroxyl, carboxyl, and epoxy groups, which increase the wettability of the GO membrane. On the other hand, the contact angles of GO-EDA, GO-BDA, and GO-PPD gradually increased, changing the hydrophobicity slowly.

There are several reasons for these. Firstly, when GO reacts with small molecule binary amines, a certain degree of reduction will occur, resulting in a large contact angle of the membrane. Secondly, as the length of the cross-linking agent molecules increases, the hydrophilicity decreases, resulting in the contact angle of GO-BDA being greater than GO-EDA. Thirdly, due to the hydrophobic structure of benzene ring, the contact angle of GO-PPD is the largest [44]. Lastly, the introduction of amine monomers increases the mass transfer resistance of water molecules through the membrane, resulting in poor wettability of the film, which leads to poor wettability of the membrane [45].

**Figure 3:**
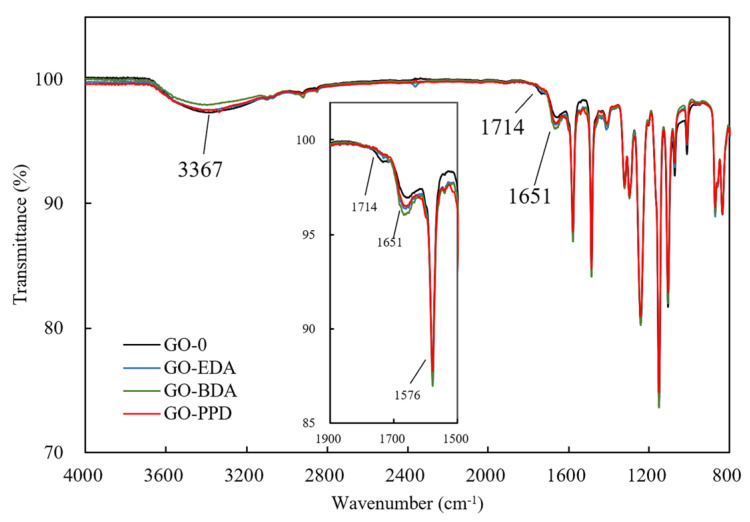
Infrared spectrogram of cross-linked GO membrane.

Figure 3 shows the infrared spectrum of GO-0, GO-EDA, GO-BDA, and GO-PPD (from 4000 to 800 cm^−1^). According to the infrared spectrum, GO-EDA, GO-BDA, and GO-PPD were weaker than GO-0 at 3367 and 1714 cm^−1^, indicating that the binary amine molecule participates in a condensation reaction with the hydroxyl and carboxyl groups of GO. In addition, the bending vibration of C-N bond was strengthened at 1651 cm^−1^, indicating a reduction reaction of GO with the diamine in some manner. The peak at 1651 and 1576 cm^−1^ indicate the bending vibration of the C-N bond [46], the carboxyl stretching vibration peak appears at 1714 cm^−1^, which represents the introduction of the hydroxyl and carboxyl groups contained in GO.

**Figure 4:**
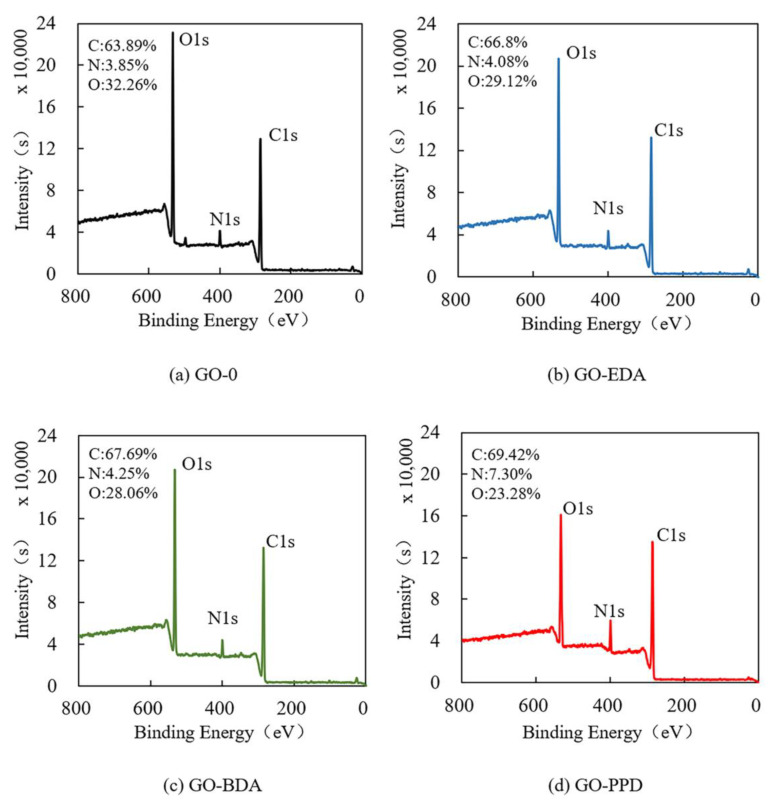
XPS full scan spectrum of: (**a**) GO-0, (**b**) GO-EDA, (**c**) GO-BDA, (**d**) GO-PPD.

Figure 4 shows the XPS full scan spectrum of GO-0, GO-EDA, GO-BDA, and GO-PPD. The O/C ratios of GO-EDA, GO-BDA, and GO-PPD were 0.44, 0.45, and 0.33, respectively, which were much lower than the ratio of 0.51 observed for GO-0. This result occurred because the oxygen-containing functional group was consumed in the cross-linking reaction, which was consistent with the results obtained from the infrared spectrum.

### 3.2. Flux of Cross-Linked GO Self-Supporting Membrane

The pressure filtration effectively overcame the swelling phenomenon of GO-0, and the relative relationship between the film layer spacings was consistent with the dry state. According to the Bragg equation, the layer spacing can be obtained, as shown in Table 2.

Since no cross-linking agent was added between the GO nano-layers, the layer spacing was minimized under pressure filtration conditions. On the other hand, the length of EDA molecule was short, so the spacing between GO-EDA layers was less than GO-BDA. As per the GO-PPD, although the carbon chains between the BDA and PPD amine groups were similar, the layer spacing of the GO-PPD was larger because of the steric hindrance effect of the benzene ring [47].

**Figure 5:**
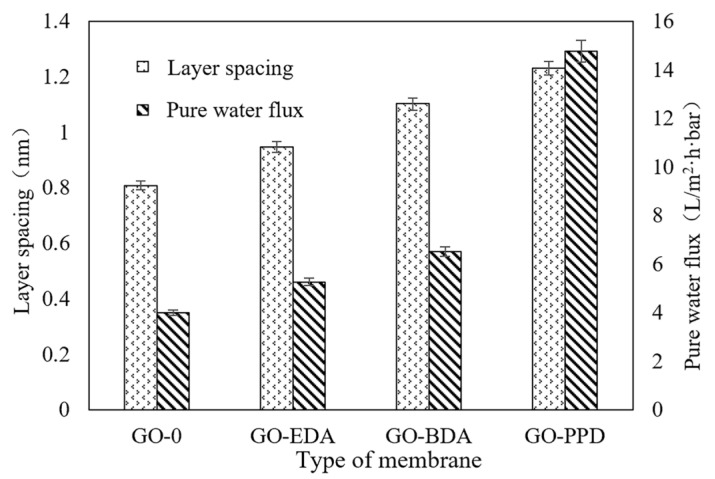
Pure water flux and layer spacing of cross-linked GO membrane.

Membrane flux is generally determined by the hydrophilicity, hydrophobicity, and pore size of the membrane. It can be seen from Figure 5 that, in the cross-linked GO membrane, the flux increased with the increased layer spacing, and the following flux trend was exhibited: GO-0 < GO-EDA < GO-BDA < GO-PPD. The results showed that the length and the properties of the cross-linking agent were the main factors affecting the flux. Adding a cross-linking agent into the GO layer can effectively increase the membrane flux while enhancing the stability of the GO membrane.

### 3.3. Adsorption of PPCPs by Cross-Linked GO Self-Supporting Membranes

The hydrophobic substances ibuprofen, gemfibrozil, and triclosan were selected as the target pollutants. The adsorption of PPCPs by cross-linked GO membrane at pH 3 was investigated.

**Figure 6:**
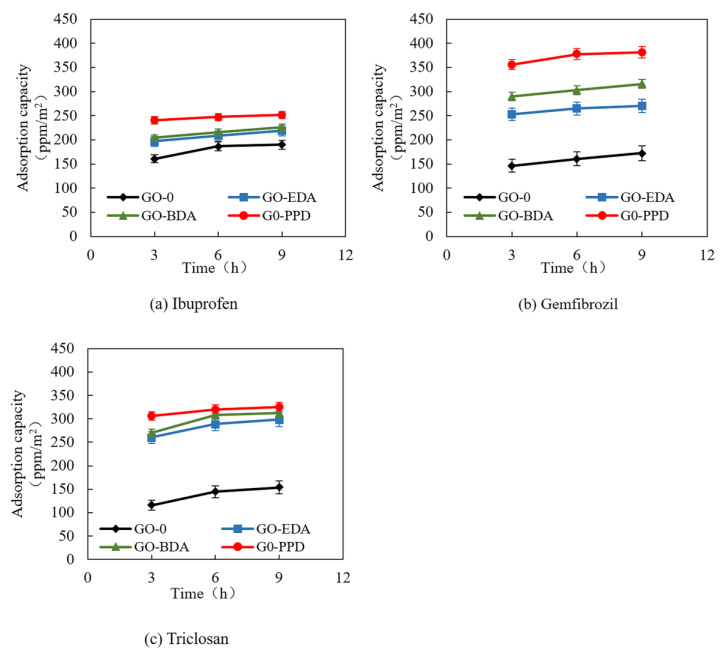
The adsorption capacity of the pharmaceuticals and personal care products (PPCPs) (**a**) ibuprofen, (**b**) gemfibrozil, and (**c**) triclosan onto the cross-linked GO membrane in the absence of pressure.

Figure 6 shows the adsorption of ibuprofen, gemfibrozil, and triclosan by GO membranes with time in the absence of pressure. The GO membrane displayed a certain adsorption effect on the PPCPs. After 3 h, the membranes reached approximately 90% of their adsorption capacity and were in a state of adsorption stability. Comparing the adsorption of GO membranes on different substances revealed that the adsorption capacity of cross-linked GO membranes for gemfibrozil and triclosan was approximately 1.5 times that of GO-0 for gemfibrozil and triclosan. However, the adsorption capacity of GO-0 for ibuprofen was slightly higher than that of gemfibrozil and triclosan. In addition, the order of adsorption of the membrane for the PPCPs was expressed as GO-0 < GO-EDA < GO-BDA < GO-PPD. Along with Figure 2, these data provided a comprehensive analysis of the hydrophilicity, hydrophobicity, and permeability of GO membranes. The adsorption of PPCPs by GO membranes is determined by the properties of the membrane and the PPCPs [29,48]. The stronger the hydrophobicity of the membrane, the weaker the permeability. The stronger the hydrophobicity of PPCPs, the higher the adsorption capacity of the GO membrane for PPCPs, according to the principle of “similar phase solubility” [49,50,51,52].

### 3.4. Removal of PPCPs by Cross-Linked Self-Supporting GO Membranes

**Figure 7:**
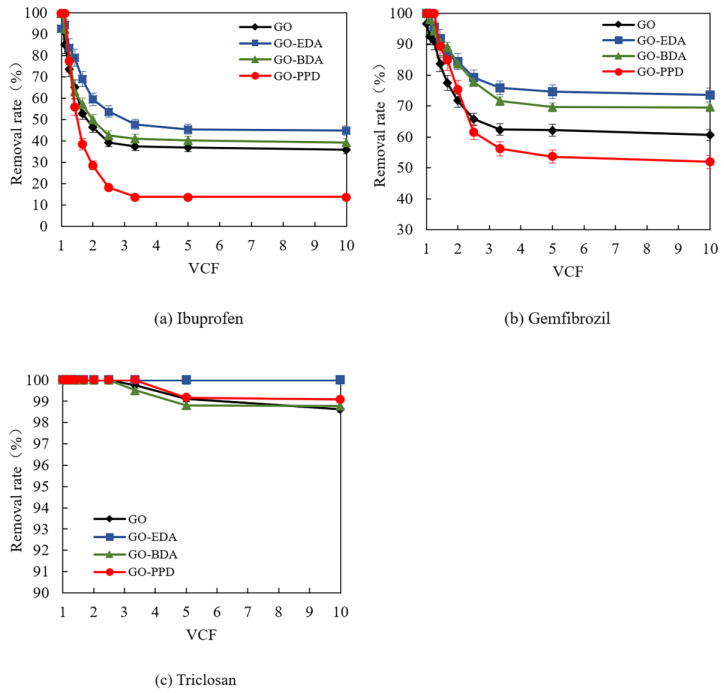
The removal rate of PPCPs: (**a**) ibuprofen, (**b**) gemfibrozil, and (**c**) triclosan by cross-linked GO membrane at pH 3.

The removal rate of the three substances at different volumentric concentration factors (VCF) in the GO film was ibuprofen < gemfibrozil < triclosan, wherein the removal rate of triclosan was 98 to 100% (Figure 7). The reason for the pattern was that when the membrane reaches the adsorption saturation of triclosan, the larger steric hindrance of triclosan fully exerts an interception effect of GO membranes. GO-EDA showed the highest removal effect in four membranes (44.9% for ibuprofen, 73.6% for gemfibrozil, and 100% for triclosan), indicating that it has the best ability to retain pollutants. In addition, the flux of GO-EDA (5.27 L/m^2^∙h∙bar) was greater than that of the GO-0 membrane flux (4.0 L/m^2^∙h∙bar), indicating that the introduction of an appropriate cross-linking agent can simultaneously increase the removal rate and the membrane flux.

Therefore, if the concentration of ibuprofen or gemfibrozil needs to be reduced, the raw material solution can be adjusted to be acidic before passing through the membrane and being removed by GO-EDA. If triclosan needs to be removed, the feed solution can be adjusted to be acidic before passing through the membrane and being completely removed by GO-PPD (14.78 L/m^2^∙h∙bar) with the highest flux.

**Figure 8:**
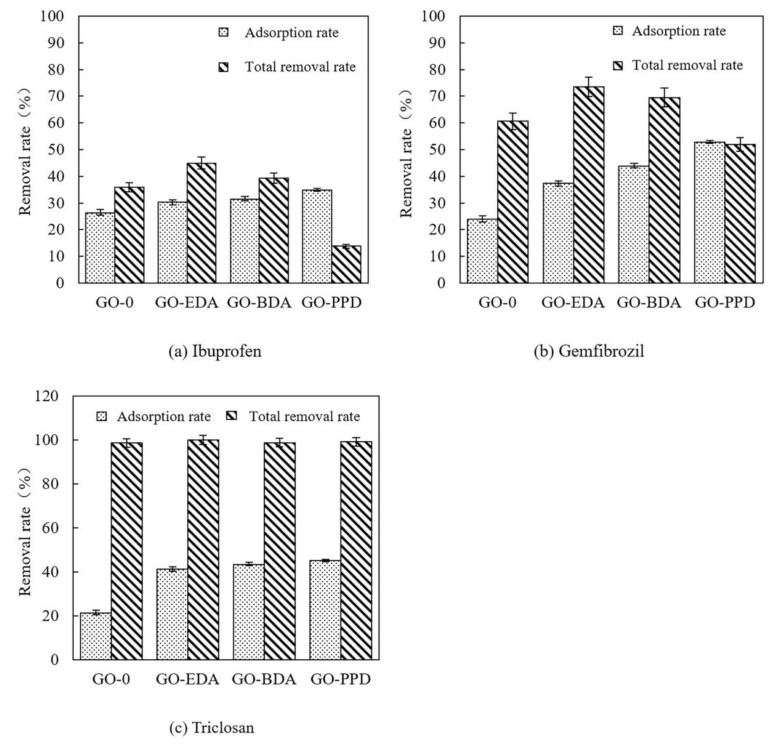
The adsorption rate and total removal rate of PPCPs: (**a**) ibuprofen, (**b**) gemfibrozil, (**c**) triclosan using the cross-linked GO membrane at pH 3.

Figure 8 shows the adsorption removal rate and total removal rate of PPCPs by GO membranes. The adsorption removal rates of ibuprofen and gemfibrozil by GO-PPD (34.8% and 52.83%, respectively) were higher than the total removal rates (13.8% and 51.93%, respectively) (Figure 8a,b). This phenomenon is attributed to the fact that when the GO-PPD reaches the adsorption equilibrium, the PPCPs adsorbed on the surface of the membrane will penetrate the pores of the membrane, so that the final apparent removal rate is the mechanical sieve removal rate of the membrane pores [53]. However, the GO-PPD layer spacing was large, and so the screening effect of substances such as ibuprofen and gemfibrozil was not significant. The removal rate of triclosan exceeded 99%, possibly because its structure contained two benzene rings and had a large steric hindrance. In addition, the hydrophobicity of Triclosan is strong. Considering both hydrophobicity and steric hindrance, triclosan shows the highest removal rate. Overall, the apparent removal rate of PPCPs by GO membranes involves the adsorption interception of the membrane surface and mechanical screening interception of membrane pores. However, adsorption has a positive effect on the removal of PPCPs only in the initial stage of filtration. In the long run, the relationship between layer spacing and steric hindrance is the main factor affecting the retention of hydrophobic PPCPs by GO membranes.

## 4. Conclusions

In this study, cross-linked self-supporting GO-EDA, GO-BDA, and GO-PPD membranes with surface density of 25 mg/m^2^ were prepared by the pressure-assisted self-assembly method. GO-EDA, GO-BDA, and GO-PPD membranes were characterized and compared with GO-0 by infrared spectroscopy, contact angle measurements, XPS, XRD, and pure water flux. The hydrophobic substances of ibuprofen, gemfibrozil, and triclosan were used as target pollutants. The adsorption and molecular sieving of PPCPs by cross-linked GO membranes at pH 3 were investigated. Through the adsorption and filtration experiments, the removal mechanism of pollutants from the membrane was further studied from the perspectives of hydrophilic and hydrophobic properties, molecular weight and substance types, combined with qualitative analysis and quantitative analysis. It can be seen from the infrared spectrogram, dynamic contact angle measurement and XPS energy spectrum that the small binary amine molecules in GO-EDA, GO-BDA, and GO-PPD have condensation reactions with the hydroxyl and carboxyl groups on GO. This can reduce the hydrophilicity and permeability of GO-0, and the longer the molecular length of the diamine, the greater the steric hindrance, and the stronger the hydrophobicity. Besides, the GO-0, GO-EDA, GO-BDA, and GO-PPD membranes were placed under a pressure filtration condition of 3 bar, and the inter layer spacing of the membranes was measured by an X-ray diffractometer. The results showed that the pressure filtration can effectively overcome the swelling phenomenon of the GO membrane, and the relative relationship between the membrane layer spacings was consistent with the dry state and positively correlated with the flux. Unlike traditional nanofiltration membranes, which rely mainly on electrostatic repulsion, the removal of hydrophobic PPCPs in the molecular state by GO membranes was mainly due to adsorption and molecular sieving. The adsorption was mainly determined by the hydrophilic and hydrophobic properties of the membrane and PPCPs [29]. The stronger the hydrophobicity of the membrane and the stronger the hydrophobicity of PPCPs, the greater the amount of PPCPs adsorbed by the membrane. The interception effect was mainly determined by the interlayer spacing between the GO membranes and the molecular weight and steric hindrance of the PPCPs. In the long term, the relationship between layer spacing and molecular weight is the main factor affecting the interception of hydrophobic PPCPs by GO membranes. The smaller the spacing of the GO membrane layers, the larger the steric hindrance and the higher the removal rate. Comprehensively considering the membrane flux and removal rate, GO-EDA removed 47.20% of ibuprofen, 73.59% of gemfibrozil, and 99.7% of triclosan under a flux of 5.27 L/m^2^∙h∙bar. GO-PPD removed 99.2% of triclosan under a flux of 14.78 L/m^2^∙h∙bar.

## Figures and Tables

**Table 1:** Characteristics of the organic compounds.

Target Compound	CAS Number	Molecular Weight	Acid Dissociation Constant (pKa)	N-Octanol/Water Partition Coefficient (logKOW)	Chemical Structural Formula
Ibuprofen	15687-27-1	206.3	4.91	3.97	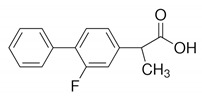
Gemfibrozil	25812-30-0	250.3	4.7	4.77	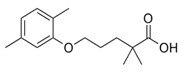
Triclosan	3380-34-5	289.5	7.9	4.76	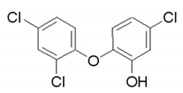

**Table 2:** Layer spacing of graphene oxide membrane.

Membrane Type	GO-0	GO-EDA	GO-BDA	GO-PPD
**Layer spacing**	0.81 nm	0.95 nm	1.10 nm	1.23 nm
